# A scoping review on ethnobotanical uses, pharmacological and toxicological profiles of traditional medicinal plants in paediatric healthcare across Sub-Saharan Africa and South Asia

**DOI:** 10.1186/s12906-026-05336-z

**Published:** 2026-03-20

**Authors:** Shikwambana Zanele Geraldin, Ramashia Shonisani Eugenia, Frans Koketso Matlakala, Nethathe Bono

**Affiliations:** 1 Department of Food Science and Technology, Faculty of Sciences, Engineering & Agriculture, University of Venda, Private Bag X5050, Thohoyandou, Limpopo Province 0950 South Africa; 2 Research and Innovation Directorate, University of Venda, Thohoyandou, South Africa

**Keywords:** Medicinal plants, Ethnomedicine, Pharmacological activity, Toxicity, Infants’ health

## Abstract

Traditional medicinal plants play a crucial role in paediatric healthcare, especially in underdeveloped countries where access to modern medicine is limited. This review synthesizes ethnobotanical, pharmacological, and toxicological data on the use of medicinal plants in treating childhood ailments. This scoping review was guided by Arksey and O’Malley framework. Different databases were utilized for searching relevant articles, and references were managed using EndNote 21. A total of 49 studies were analysed, including 14 ethnobotanical, 18 pharmacological, and 17 toxicological studies. Ethnobotanical results revealed 54 plant species from 33 families, with Fabaceae having the most species, and South Africa contributing the most studies. The plant's leaves were most commonly used, and the remedies were prepared as decoctions and administered orally. Some of the ailments these plants treated included respiratory and gastrointestinal infections. Pharmacological analyses confirmed antimicrobial, anticancer, antifungal, antioxidant, and anti-inflammatory properties in 18 plants reviewed, notably *Annona senegalensis* Pers*., Punica granatum* L, and *Curcuma longa* L, justifying their traditional use. However, toxicological results showed that only 10 of the 18 pharmacologically analyzed plants showed some level of toxicity, especially in brine shrimp assay or at high doses. In conclusion, the review highlights the potential of traditional medicinal plants to be both therapeutic and toxic in children. Therefore, future studies are recommended to research the standardization of remedy preparation and dosages and targeted pediatric toxicity.

## Introduction

Globally, 40–99% of the populations in WHO Member States use traditional, complementary, and integrative medicine [1]. Many underdeveloped countries rely on traditional medicinal plants as the primary healthcare because of their affordability, accessibility, and cultural acceptance [2]. Traditional medicinal plants have been used for generations across various cultures to treat various ailments, including those affecting infants and children [3, 4]. Furthermore, in some cultures, traditional medicinal plants are believed to possess powers to expel evil spirits that may cause illness in a child [5, 6]. In South Africa, one of the traditional ceremonies performed by the Xhosa tribe on newborn babies involves tying a bag filled with various herbs and medicinal plants around the baby's neck or stomach to chase away evil spirits [7]. Plants such as *Siphonochilus aethiopicus* and *Commelina diffusa* are reported to possess such powers [8, 9].

While in the area of the Venda tribe (South Africa), babies as young as 2–3 months old are fed with various medicinal plants, such as *Annona senegalensis* Pers. and *Ximenia Caffra* Sond*.* and *Piliostigma Thoningii* (Schumach.) Milne-redh., mixed with weaning food to strengthen their immune system [10]. Moreover, such practices are also observed in India, where *Betula utilis*, *Prunus cerasoides* Buch. and *Trachyspermum ammi* L. are made into amulets that are hung around the neck, waist or arm of the child for their well-being [11]. The traditional uses of traditional medicinal plants prompted an increase in scientific data exploring their pharmaceutical properties [12, 13]. The data revealed that most, if not all, plants contain promising bioactive compounds, including alkaloids, flavonoids, phenolics, saponins, and tannins, with antidiarrheal, antimicrobial, antiviral, antifungal, anti-inflammatory, and antipyretic properties.

Despite the widespread use of traditional medicinal plants in paediatric healthcare, there is limited scientific data validating their efficacy and safety, and elucidating their mechanisms of action [14]. Furthermore, most traditional knowledge is passed down orally from generation to generation, making it difficult to standardise the preparation and dosages of these plants, especially for vulnerable populations such as neonates and infants [15]. Infants' metabolic system is not fully mature, making this group more susceptible to the toxic effects of bioactive plant compounds. Side effects in infants range from neurotoxicity, organ failures, and in extreme cases, death [16]. Moreover, in cases of herbal intoxication, admission to hospitals suggests there is an urgent need to assess the toxicological profiles of these plants [16]. Therefore, understanding the balance between therapeutic benefit and potential harm is important, especially for infants, because their safety threshold is lower than that of adults with mature metabolic systems [17].

This review is therefore timely and essential, aiming to bridge the gap between traditional knowledge and modern scientific validation. By systematically evaluating existing studies on the ethnobotanical uses, pharmaceutical and toxicological profiles of medicinal plants used in paediatric care (infants and children), the review will provide a consolidated evidence base to inform clinical practice and guide further research on the safe integration of traditional medicine in child healthcare systems.

## Methods

### Research approach

The researchers adopted scoping review [18] to scope the existing literature on the ethnobotanical uses, pharmacological and toxicological profiles of traditional medicinal plants in paediatric healthcare. Scoping review as a framework has five steps that researchers must follow in order to derive the desired goal [18], which are discussed in detail below:

#### Step 1: identifying the research question

The first step required researchers to develop a research question that is linked to the study objective or aim. Arksey and O’Malley [18] advise that, to get this step right, several frameworks can be adopted to ensure the review question is clear and well-defined. To do so, the researchers adopted the Population, Concepts and Context (PCC) framework to develop the research question. The research question developed was “*What are the ethnobotanical uses, pharmacological activities, and toxicological profiles of traditional medicinal plants used in paediatric healthcare?”.*

#### Step 2: identify relevant studies/literature

The researchers relied on various databases such as Web of Science, ScienceDirect, Sabinet African Journals, and PubMed. The search utilized a combination of keywords and Boolean operators, truncations, and Medical Subject Headings to widen the search. The developed search string was: (“traditional medicinal plants" OR "indigenous medicinal plants” AND “pharmacological activity” OR bioactivity AND “toxicological profile” OR toxicity AND infant OR pediatric OR “child health”). The above search string was used in the selected databases, and the faculty librarian was requested to assist in aligning to each database’s advanced search, as it is suggested for review studies [19] (Appendix 1). Before exporting articles to a reference manager, EndNote 21, we developed inclusion and exclusion criteria (Table 1).

**Table 1:** Eligibility criteria

Criterion	Inclusion	Exclusion
Target Population	Infants/children using traditional medicinal plants for their healthcare needs	People who are not regarded as children are excluded
Design	Qualitative, Quantitative, Mixed methods, experimental, observational, and case studies	Editorials, non-peer-reviewed articles, protocol, preprints, and review studies
Phenomenon of interest/Outcome	Studies reporting on specific plant species utilized for children, including details on preparation, dosage, and pharmacological or toxicity testing results	Studies that do not report on plant species utilized for children, including details on preparation, dosage, and pharmacological or toxicity testing results
Language	Studies published in English	Studies in languages that cannot be translated or are inaccessible, to maintain consistency and manageability
Period	Studies published between January 2014 and June 2025 will be reviewed to ensure that the review captures recent and relevant data pre-, during, and post-COVID pandemic	Studies published outside this period (before 2014 and after June 2025)

#### Step 3: study selection

**Fig. 1:**
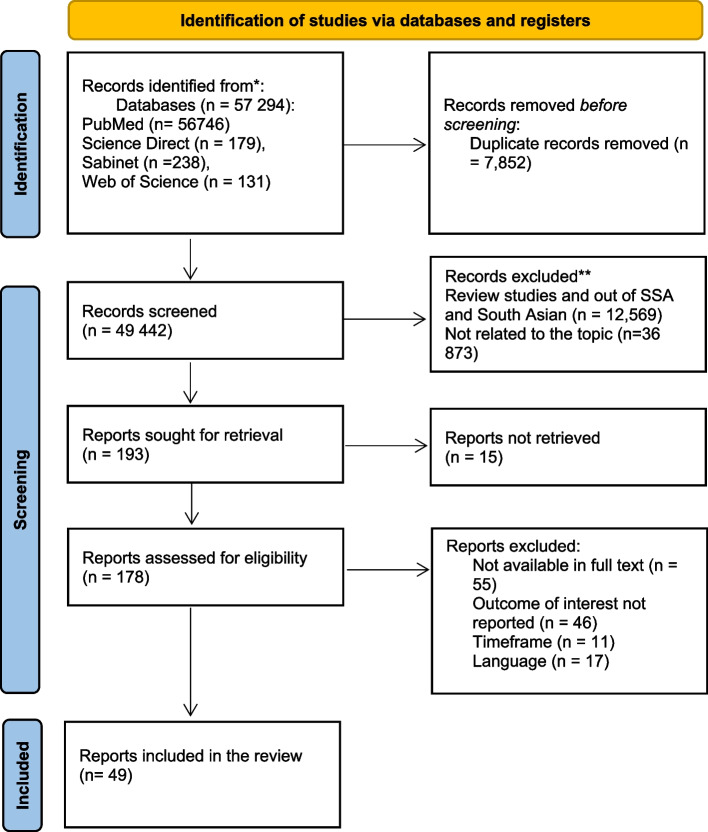
PRISMA flowchart showing the results of the literature search and the method used to select relevant documents. Source: Page et al. [20]

In order to perform screening, articles that met the inclusion criteria were exported to the reference manager, EndNote 21. Firstly, all articles from various databases were combined in the reference manager to remove duplicates. Thereafter, three reviewers (BN, ZS, and RS) performed title and abstract (T&A) screening, with one reviewer (FK) serving as the mediator and monitor of the process. After performing T & A screening on EndNote 2.1, articles that met the inclusion criteria were moved to another group titled full-text Screening. We then applied the option “retrieve PDFs” in the group “full-text screening” (see Fig. 1 for the summarised process).

#### Step 4: data charting

After gathering all the articles, a data chart was created in Microsoft Word to facilitate data synthesis. Articles not meeting the inclusion criteria during full-text screening were removed from the reference manager group tab, and those meeting the inclusion criteria were added to the data charting. The data chart had the following headings: years of publication, countries of publication, plants common names, plant parts used for making remedies, mode of preparation, dosage, administration, disease treatment/management, pharmacological activity, and toxicological profiles.

#### Step 5: collating, summarising, and reporting

The data on the data chart was summarized on each of the developed headings as indicated in step 4. To be able to report on the findings, we relied on content analysis. The findings were reported in data charts and tables in the results section (Tables 4 and 5). Moreover, before producing this article, we used the checklist study (PRISMA 2020) for rigor in the study.

Figure 1 shows that the search identified 57 294 records, which were downloaded into Endnote. After removing duplicates, 49 442 records were left. After screening titles and abstracts, 178 articles were eligible for full-text screening. Thereafter, screening of the full articles was conducted; 129 articles were excluded because full articles were not available, the focus of the study was not related to children's health, leaving 49 articles.

## Results

### Ethnobotanical results

**Fig. 2:**
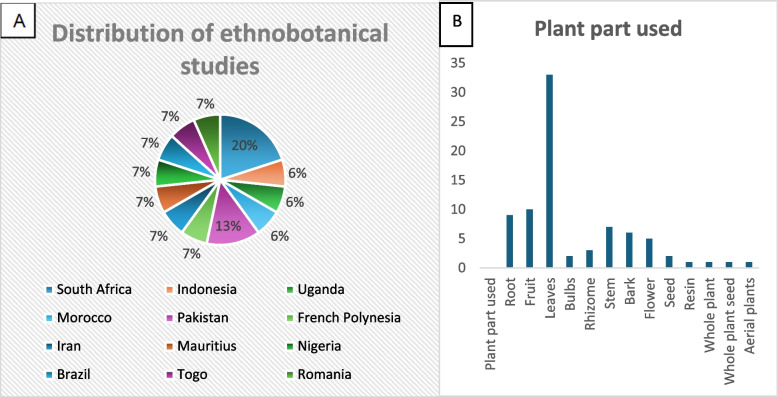
**A** Distribution of ethnobotanical studies globally. **B** Frequency index of part of the plant used for medicinal purposes

From the 14 studies, geographically, the distribution of studies revealed that South Africa contributed the most, accounting for 20% of the publications [2, 8, 21]. This was followed by Pakistan with 13% [30, 31], while the remaining studies were distributed among other countries such as Nigeria, Brazil, Indonesia, and Mauritius, each contributing less than 7% [23–25, 28] (Fig. 2A).

Furthermore, data charting identified 54 medicinal plant species from 33 botanical families used in child health management [2, 3, 8, 22–31] (Table 2). The Fabaceae family was the most cited, comprising eight species commonly used to treat diarrhea, measles, dermatitis and malaria across different countries [8, 22–25, 29, 30] (Table 2).

**Table 2:** Plant families most utilized for children`s healthcare

Family	Countries	Common Uses	Citation count
Fabaceae	Nigeria, South Africa, Uganda, Brazil, Pakistan, Indonesia	Diarrhea, measles, dermatitis, malaria	8
Asteraceae	Uganda, South Africa, Mauritius, Iran	Measles, fontanelles, stomach issues	6
Amaryllidaceae	Nigeria, Indonesia, Brasil	Dermatitis, cough, stomachache	4
Euphorbiaceae	Nigeria, South Africa	Dermatitis, constipation	4
Lamiaceae/Verbenaceae	Brasil, South Africa, Morocco	Fever, cough, antispasmodic	4

Leaves, fruits, and roots were the most commonly used plant parts across all studies, and they are frequently prepared as decoctions, infusions, or powders, and they are mostly administered orally and topically [3, 21–24, 26–28] (Fig. 2B). These preparations were used to treat a broad range of childhood illnesses, predominantly respiratory, gastrointestinal, and dermatological conditions. Additionally, in South Africa, these plants were reported for managing culturally defined ailments such as bulging or sunken fontanelle and umbilical cord complications [2, 8, 21], which are not commonly addressed in conventional biomedical literature.

### Pharmacological activities

India stood out as a pioneer, contributing 16% to these studies [32, 34, 59, 60], followed by Nigeria contributing 11% [12, 37]. Antimicrobial activities were the most frequently reported on plants such as *Momordica charantia* L., *Ammodaucus leucotrichus* L., *Anchomanes difformis* (Blume) Engl., *Annona senegalensis*, *Xylopia aethiopica* (Dunal) A.Rich., *Bulbine frutescens* (L.) Willd., *Cymbopogon citratus* (DC.)Stapf., *Oxalis corniculate* L., *Citrullus colocynthis* L., and *Parkia biglobosa* (Jacq) Benth. [13, 34, 36, 37, 42, 46, 48, 52, 56]. Antioxidant properties were also commonly reported in plants such as *Curcuma longa* L., *Punica granatum* L., *Fumaria officinalis* L., and *Bulbine frutescens* (L.). Willd., *parkia biglobosa* (Jacq) Benth.*,* and *Vernonia amygdalina* Delile [12, 44, 46, 48, 50, 54]. Anti-inflammatory effects were observed in *Aloe vera* (L.) Burm.F. *Annona senegalensis* Pers., and *Ammodaucus leucotrichus* L*.* [32, 36, 42], and antifungal activity was primarily reported in *Syzygium aromaticum* L. [63]. Anticancer activity was noted in *Capsicum frutescens* L., *Mormordia charantia* L., *Xylopia aethiopica* (Dunal) A.Rich., *Zingiber officinale* Schumm*.*, *Punica granatum* L. and *Tetrapleura tetraptera* [34, 37, 59, 65, 66]. The pharmacological tests conducted in these studies were preclinical in vitro assay. Antimicrobials were evaluated using disk diffusion and minimum inhibitory concentration (MIC); DPPH/FRAP for antioxidants; NF-κB inhibition or COX-1/2 for anti-inflammatory; and MTT/SRB for anticancer.Pharmacological findings were also linked to ethnobotanical applications, supporting their traditional use in treating infections in children (Table 3).

**Table 3:** Correlation between pharmacological findings and traditional uses

Scientific name	Traditional uses	Pharmacological activities
*Aloe vera* (L.) Burm. F	Chicken pox, measles	Anti-inflammatory
*Ammodaucus leucotrichus* L	Nausea, vomiting, dysentery, regurgitation	Antimicrobial, Anti-inflammatory
*Xylopia aethiopica* (Dunal). Rich	Dermatitis, malaria	Antimicrobial, anticancer, antioxidant
*Vernonia amygdalina* Delile	Malaria	Antioxidant
*Annona senegalensis* Pers	Sunken/building fontanelle	Anti-microbial, anti-inflammatory
*Ayapana triplinervis* (M.vahl) R.King & H.Robison	Vomiting, stomach pains, diarrhea, colitis	Antioxidant
*Bulbine frutescens* (L) Willd	Sunken fontanelle, umbilical cord, body rash, sores, urinary tract infections	Antioxidant, antimicrobial
*Curcuma longa* L	Diarrhea, boils, fever	Antioxidant
*Cymbopogon citratus* (DC) Stapf	Dermatitis	Antibacterial
*Fumaria officinalis L*	Jaundince	Antioxidant
*Parkia biglobosa* (Jacq) R.Br. exg.Don	Diarrhea, oral thrush	Antimicrobial, antioxidant
*Syzygium aromaticum*	Dermatitis	Antifungal
*Tetrapleura tetraptera* (Schum Z & Thom)	Dermatitis	Anticancer
*Zingiber officinale Rosc*	Cold, asthma	Anticancer, antibacterial

### Toxicological evaluation

Toxicological data varied across the reviewed studies. Of the 18 plants, 10 showed some form of toxicity, either through acute, sub-chronic, or brine shrimp lethality assays [33, 35, 36, 38–41, 43, 45, 47, 49, 51, 53, 55, 57, 58, 62, 64, 65]. Toxic plants included *Ammodaucus leucotrichus* L., *Capsicum frutescens* L., *Anchomanes difformis* (Blume) Engl., *Xylopia aethiopica* (Dunal) A. Rich., *Fumaria officinalis* L., *Ayapana triplinervis* (Vahl.), *Oxalis corniculate* L., *Parkia biglobosa* (Jacq) Benth., *Citrullus colocynthis* L., *Syzygium aromaticum* (L.) Merr., and *Tetrapleura tetraptera* (Schumm.) [38–40, 45, 49, 55, 57, 58, 64]. Reported toxic effects occurred at varying doses; for example, *A. leucotrichus* L. oil was toxic at 520–570 mg/kg body weight in mice after 14 days of exposure [38], and *X. aethiopica* (Dunal) A. Rich. at 1000 mg/kg in rats after 28 days [40]. The Brine Shrimp Lethality Test indicated toxicity in *A. triplinervis* (Vahl) and *Oxalis corniculate* L., with LC_50_ values of 18.86 ppm and 156 µg/ml, respectively, after 24 h [45, 57]. These findings indicate potential cytotoxicity at relatively low concentrations. Conversely, eight plants (*Aloe vera* (L.) Burm. F., *Curcuma longa* L., *Vernonia amygdalina* Delile., *Punica granatum* L., *Zingiber officinale* Roscoe., *Momordica charantia* L., *Bulbine frutescens* (L.) Willd.*, Annona senegalensis* Pers., and *Cymbopogon citratus* (DC.) Stapf. showed no observable toxicity at therapeutic doses [33, 35, 39, 41, 43, 47, 51, 53, 62]. For instance, *C. longa* showed no sub-chronic toxicity at doses up to 1000 mg/kg body weight in rats over 90 days [51] (Table 4).

**Table 4:** Indigenous uses of traditional medicinal plants in pediatric healthcare

No	Author	Country	Family	Scientific name	Common name	Plant part (s) used		Method of preparation	Dosage and administration	Medicinal use
1.	Mashile et al. [2]	South Africa	Agapanthaceae	*Agapanthus africanus* (L.) Hoffmanns	Cape agapanthus		R	Decoction	Oral; bath; in hail smoke	Resuscitation, nail-biting
			Anacardiacea	*Sclerocarya birrea* (A.Rich.) Hochst	Marula		Fr	-	Tie dry fruit around a child`s neck or hands	Hiccups
			Annonacea	*Annona senegalensis* Pers	Africa custard-apple		R	Decoction	Oral, give in small quantities or with a formula	Sunken or bulging fontanelle
			Facaceae	*Senna petersiana* (Bolle) Lock	Coffe senna		R	-	Oral, given in small quantities	Colic, sunken or bulging fontanelles
2.	Rankoana [21]	South Africa	Verbenaceae	*Lippia javanica* (Burm F.)	Fever tea		L	Infusion	-	Cough
			Asphodelaceae	*Aloe ferox* Mill	Bitter aloe		L	Infusion	-	Chickenpox, measles
			Asteraceae	*Bidens pilosa* L	Blackjack		L	Poultice	-	Pulsating fontanelle
			Euphorbiaceae	*Ricinus communis* L	Castor bean		L	Infusion	-	Constipation, stomachache, red mark on the back of neck
			Solanaceae	*Datura stramonium* (Linn.)	Jimsonweed		Bu	-	Rub on dermal incisions made in the chest	Cough, flu
3.	Ndhlovu et al. [8]	South Africa	Asparagaceae	*Euphorbia prostrata* Aiton	-		Rh	Decoction or enema	Orally (as needed)	Constipation and phlegm
-			Commelinaceae	*Commelina diffusa* Burm.f	Spreading dayflower		Rh/Bu	Decoction	Orally 2 × daily	Umbilical cord, purgative the child, preventing evil spirits and weak child
			Fabaceae	*Elephantorrhiza elephantina* (Burch) Skeels	Eland's bean		R	Maceration or poulice	Orally and topical (3 × daily)	Infective eczema, diarrhoea, ulcer, burns and measles
			Scrophulariaceae	*Aptosinum elongatum* Eng	-		St	Infusion	Orally 3 × daily	Umbilical cord, muscle fits, measles, bladder inflammation, weight and appetite
			Xanthorrhoeaceae	*Bulbine frutescens* (L.) Willd	-		Rh/Bu; R	Infusion, maceration	Topical and orally (2 × daily)	Sunken fontanelle, umbilical cord, body rash, sores, and urinary tract infection
4.	Nalumansi et al. [22]	Uganda	Amaranthaceae	*Chenopodium opulifolium* Schrad. ex W.D.J.Koch & Ziz	Seaport goosefoot		L	Decoction	-	Measles
			Asteraceae	*Vernonia amygdalina* Delile	Bitter leaf		L	Decoction	-	Malaria
			Cucurbitaceae	*Kedrostis foetidissima* (Jacq.) Cogn	-		L	Prepared as accompaniment of staple or cooked with silver fish	Oral	Appetite Boosting
			Fabaceae	*Albizia coriaria* Oliv	-		B	Decoction	-	Malaria
			Verbenaceae	*Lantana camara* L	Common lantana		L	Decoction; Crushed in water	-	Malaria
5.	Erinoso et al. [23]	Nigeria	Amaryllidaceae	*Allium ascalonicum* L	Alubosa elewe (Yoruba), Shallot		L	Mixed with *Syzygium aromaticum* fruit, *Picralima nitida* seed, *Smilax kraussiana* root, and *Griffonia simplicifolia* root. Decoction with pure water	Oral (2–3 teaspoonful) 3 × daily; for bath	Dermatitis
			Annonaceae	*Xylopia aethiopica* (Dunal) A. Rich	Eru-alamo (Yoruba)		Fr	Mixed with *Aloe vera* leaf, *Euphorbia laterifolia* stem, *Tetrapleura tetraptera* fruit and *Senna alata leaf* leaf. The plants are ground and worked into the black soap	For bath. Coconut oil is used as cream to massage the affected area	Dermatitis
			Euphorbiaceae	*Euphorbia laterifolia* Schum. & Thonn	Enu-opiri (Yoruba)		L	Mixed with *Allium sativum* bulb, *Piper guineense* fruit, *Parkia biglobosa*, and *Tetrapleura tetraptera* fruit. Decoction with pure water	For bath only	Dermatitis
			Fabaceae	*Tetrapleura tetraptera* (Schum. & Thonn.) Taub	Aidan (Yoruba)		Fr	Mixed with *Euphorbia hirta* leaf*, Cymbopogon citratus* leaf, *Dennettia tripetala* seed*,* and *Lantana camara* twig. Decoction with pure water. For bath only	For bath only	Dermatitis
			Fabaceae	*Senna alata* (L.) Roxb	Asunwon (Yoruba)		L	Squeeze the leaf to extract juice and apply juice to affected area	-	Dermatitis
			Poaceae	*Cymbopogon citratus* (DC.) Stapf	Ewe tea (Yoruba), Lemon grass		L	Mixed with *Citrus aurantifolia* fruit and *Khaya grandifoliola* stem bark. Decoction with pure water	Oral (2–3 teaspoonfuls) 3 × daily. Coconut oil is used as cream to massage the affected area	Dermatitis
6.	Lemos et al. [24]	Brazil	Amaryllidaceae	*Allium ascalonicum* L	Shallot			Infusion	Tea (infusion), lambedor	Sore throat, coughing with secretions, Cough, hoarseness
			Fabaceae	*Cajanus cajan* (L.) Millsp.	-		L	Infusion or decoction (tea)	Tea, lambedor	Bronchitis, colds, sore throat, fever, cough
			Lamiaceae	*Ocimum basilicum* L	Basil		L	Infusion or decoction (tea)	Tea, mouthwash; gargle	Cough, flu, bronchitis, sore throat
				*Plectranthus* *Amboinicus* (L.) Spreng	French thyme		L	Infusion	Tea, juice, lambedor	Cough, sore throat, bronchitis
				*Hyptis suaveolens* (L.) Poit	Chia		L	Infusion	Tea, bath in the head	Flu, fever, nasal congestion
			Myrtaceae	*Eucalyptus globulus* Labill	Blue gum		L	Infusion or decoction (tea)	Tea, inhalation	Flu, nasal congestion, sinusitis, fever, cough
7.	Nababan & Hakim [25]	Indonesia	Amaryllidaceae	*Allium cepa* L	Onion		-	-	-	Stomachache, boils
				*Allium sativum* L	Garlic		-	-	-	Flatulence, boils
			Clusiaceae	*Garcinia mangostana* L	-		-	-	-	Thrush
			Fabaceae	*Mimosa pudica* L	Sleepy plant		-	-	-	Fever, urinary pain
			Piperaceae	*Piper betle* L			-	-	-	Colds
			Thymelaeaceae	*Phaleria macrocarpa* (Scheff.) Boerl			-	-	-	Fever, boils, smallpox
			Zingiberaceae	*Curcuma longa* L	Turmeric		-	-	-	Diarrhea, boils, fever
				*Zingiber officinale* Rosc	Ginger		-	-	-	Cold, asthma
				*Kaempferia galanga* L			-	-	-	Tonsillitis, flatulence
8.	Chassagne et al. [26]	French Polynesia	Anacardiaceae	*Spondias dulcis* L			Fr; L	Decoction/Infusion	Bath, massage, oral, gargling	Cough
			Annonaceae	*Annona muricata* L	Soursop		L	Decoction (alone or polyherbal)	For bath, local application, massage, water spraying, oral	Restlessness, irritability
			Malvaceae	*Hibiscus rosasinensis* L. “Carnation”	Chinese hibiscus		F; L	Decoction	Bath, local application, massage, oral, water spraying, Steam Bath	Restlessness, irritability,
			Poaceae	*Saccharum officinarum* L	-		L; St	Decoction/infusion	For bath, local application, Massage, water spraying, oral	Restlessness, irritability
			Rubiaceae	*Gardenia taitensis* DC	Tahitian gardenia		L; F	Decoction/infusion	For bath, local application, massage, water spraying, oral, smell	Restlessness, irritability
			Rutaceae	*Citrus x aurantiifolia* (Christm.) Swingle	Key lime		Fr	Infusion	Orally	Detoxifying agent
9.	Said et al. [27]	Morroco	Apiaceae	*Ammodaucus leucotrichus* L	Wooly cumin		Fr	Infusion	Taken as an infusion; for Bath	Dysentery, nausea, regurgitation, vomiting
				*Carum carvi* L	Meridian fennel		Fr	-	-	Carminative, stomachic, antispasmodic pediatric antidiarrheal
				*Foeniculum* *vulgare* Mill	Fennel		Fr	-	-	Treatment of the gastrointestinal discomfort, galactagogue, carminative and pediatric antispasmodic
				*Pimpinella anisum* L	Anise		Fr	-	-	Appetizer, cholagogue, stomachic, also used against aerophagia, difficult digestion and as pediatric antispasmodic
			Verbenaceae	*Lippia citriodora* (Paláu) Kunth	Fever tea		L	-	-	Pediatric antispasmodic, eupeptic sedative
10.	Basati et al. [3]	Iran	Asteraceae	*Cichorium intybus* L	Blue daisy		R; St; L	-	-	Jaundice
			Brassicaceae	*Descurainia Sophia* (L.) Webb ex Prantl	Flixweed		S	-	-	Jaundice
			Combretaceae	*Astracantha adscendens* (Boiss. & Hausskn.) Podlech	Persian manna		Re	-	-	Jaundice
			Papaveraceae	*Fumaria officinalis* L	Fumitory		L	-	-	Jaundice
			Polypodiaceae	*Adianthum capillus-veneris* L	-		F; St; L	-	-	Jaundice
11.	Mahomoodally & Sreekeesoon [28]	Mauritius	Asteraceae	*Ayapana triplinervis* (M.Vahl) R.King and H.Robinson	Water hemp		Aerial parts	Decoction	Orally (3 × daily, for 1 week)	Vomiting, diarrhoea, stomach pain, colitis
			Asteraceae	*Matricaria chamomilla* L	Chamomile		F	Decoction of whole plant	Orally (2 × or 3 × daily, for 1 week, can be extended for 2–3 weeks)	Colic
			Lamiaceae	*Plectranthus* *Madagascariensis (*Pers.) Benth. var. madagascariensis	Thicket coleus		L	Maceration, juice is warmed with honey and fresh lemon	Orally (2 × daily for 1 week)	Cough and flu, bronchitis, asthma
			Meliaceae	*Azadirachta indica* A. Juss	Neem		L	Decoction	Herbal bath followed by application of green turmeric	Vomiting, diarrhoea, stomach pain, colitis
			Piperaceae	*Piper betle* L	Betel		L	Maceration, juice is warmed with honey and fresh lemon	-	Cough and flu, bronchitis, asthma
			Poaceae	*Cymbopogon citratus* (DC. ex Nees) Stapf	Lemon grass		Wp	Decoction	Orally (7–10 Days), ginger, sugar and fresh lemon drops, is often added to enhance the preparation	Cough and flu
12.	Koukoura et al. [29]	Togo	Celastraceae	*Maytenus senegalensis* (Lam)Exell	Arto negro (Spanish)		L; St; B	Decoction, infusion	-	Diarrheas
			Combretaceae	*Pteleopsis suberosa* Engl.&Diels	-		L; R	Decoction, Powder	-	Diarrheas, oral thrush
			Fabaceae	*Parkia biglobosa* (Jacq) R.Br.exG.Don	African locust bean		L; St; B; R	Decoction, powder	-	Diarrhea, oral thrush
			Meliaceae	*Khaya senegalensis* (Desv.) A.Juss	African mahogany		St; B	Decoction	-	Diarrheas
13.	Shaheen et al. [30]	Pakistan	Fabaceae	*Cassia fistula* Herbb. ex Oliv	Indian laburnum		Fr, F	Raw, powder, decoction	Oral	Abdominal pain, constipation, cough, diphtheria, hepatitis
			Nitrariaceae	*Peganum harmala* L	Wild rue		Ws	Decoction, Raw form	Decoction of whole plant is used as gargling; seeds are given with water once a day, whole plant dipped in mustard oil and massage it on child body for 1 month	Sour throat, abdominal pain, hepatitis
			Nyctaginaceae	*Boerhavia diffusa* Engelm. & A.Gray	Spreading hogweed		R	Raw	Necklace worn by patients. As the disease is cured, necklace become longer and the patient is comforted when the necklace length reaches the navel	Hepatitis
			Plantaginaceae	*Plantago ovata* Phil	Blond plantain		S	Raw form	Oral, seed and husk soaked in water	Dysentery Constipation
14.	Shariat et al. [31]	Pakistan	Meliaceae	*Azadirachta indica* A. Juss	Neem		B	Decoction	1–2 drops of bark decoction dropped in ear and nose then olive oil is mixed with bark and massage on the affected area	Earache, scabies
			Oleaceae	*Olea europaea* L	Wild olive		L	Boil in water	Oral	Throat infection
			Pinaceae	*Pinus roxburghii* Sarg	Chir pine		B	Powder	Grind it and then sprinkle on the affected area	Wounds
			Tamaricaceae	*Tamarix dioica* Roxb. ex Roth	-		B	Powder	Grind it and sprinkle powder on the affected area	Wounds, groin region infection

^*^(-) not specified, *Wp* Whole plant, *Ws* Whole plant seed, *B* Bark, *Bu* Bulb, *L* Leaf, *Re* Resin, *Rh* Rhizome, *R* Root, *F* Flower, *Fr* Fruit, *S* Seed, *St* Stem

## Discussion

The strengths of this review lie in its vigorous assessment of research on ethnobotanical surveys, pharmacological and toxicological profiles of traditional medicinal plants for infant health care-related illness. Literature revealed an increase in reliance on traditional medicinal plants for infant diseases [29]. The ethnobotanical results indicated that South Africa and Pakistan, were the leading countries in this field, revealing they have deeply rooted traditional medicine practices and an increase in academic interest in preserving and validating indigenous knowledge through research [2, 8, 21, 30, 31]. This also demonstrates that traditional medicinal plants remain a critical resource in paediatric healthcare, especially in developing countries where access to formal healthcare is limited [2, 29]. However, geographic bias favours nations with funding and infrastructure while underrepresenting the biodiversity of Sub-Saharan Africa and other part of the world. It also delays validation of ethnopharmacological claims for plants, increases the risks to public health posed by unproven remedies, and impedes the development of affordable pediatric healthcare solutions [29]. The representation of 12 countries with scientific publications on medical plants used for infant health care is a positive effort towards standardization of the plants when it comes to preparation, dosages, administration, and illnesses treated. However, geographic bias favours nations with funding and infrastructure while underrepresenting the biodiversity of Sub-Saharan Africa due lack of the latter. It also delays validation of ethnopharmacological claims for plants, increases the risks to public health posed by unproven remedies amid antibiotic resistance, and impedes the development of affordable pediatric healthcare solutions.

Among the plants reviewed in this study, the Fabaceae family was the most widely used across countries, followed by Asteraceae. This may be influenced by its abundance, accessibility, and phytochemical richness, thereby aiding its nutritional and therapeutic uses [67]. The plant part most commonly used across all studies was leaves. The increases in the use of leaves documented in this study indicate the need to easily access and practice sustainable harvesting when harvesting these plants. Different plant parts are believed to possess distinct therapeutic properties, and leaves have been reported to contain more phytochemicals than other plant parts [68]. The results indicate that these various plant parts are commonly prepared as decoctions, infusions, or powders. These preparation methods are also known to influence the phytochemical composition and, subsequently, the therapeutic potential of plant-based remedies [67]. Infusions and decoctions, in particular, facilitate the extraction of water-soluble bioactive compounds, such as flavonoids, tannins, alkaloids, and glycosides, which may contribute to the plants' antimicrobial, anti-inflammatory, or immunomodulatory properties [69, 70] . Administration of these preparations is primarily oral and topical, with the route of administration often dictated by the type and location of the ailment. Oral administration is typically used for systemic conditions, such as gastrointestinal disorders and respiratory infections, while topical applications are used for skin problems. This dual approach reflects a practical understanding of pharmacokinetics within traditional healthcare systems, despite the lack of formal scientific validation.

The review further underscores the extensive use of medicinal plants in treating prevalent paediatric health issues. Conditions such as respiratory infections and gastrointestinal disorders are leading causes of morbidity and mortality among children in low-resource backgrounds, and the reliance on traditional remedies highlights both the accessibility and perceived efficacy of these treatments. Of particular interest is the treatment of culturally defined paediatric conditions, such as bulging fontanelle, often interpreted in traditional medicine as a sign of underlying spiritual or physiological imbalance [2]. The continued use of plant-based remedies for such conditions not only illustrates cultural resilience but also reflects an integrated health belief system where illness is viewed through both biological and socio-cultural lenses.

The pharmacological results from this review provide scientific support for ethnomedicinal claims. Antimicrobial, antioxidant, anticancer, anti-inflammatory, and antifungal activities of these plants are justified by the abundance of bioactive compounds such as flavonoids, tannins, phenolic, alkaloids, saponins, and essential oils [34, 36, 37]. The compounds are known to inhibit microorganisms and mitigate oxidative stress, which contributes to chronic inflammation and impaired immune function in children [32]. It is important to highlight that India followed Nigeria were the leading contributors to pharmacological data in this review. This trend may be due to these countries' strong ethnobotanical research networks and policy support for traditional medicine integration into public health systems. However, this representation also reveals a major limitation: a clear underrepresentation of pharmacological research from other ethnobotanically rich regions, such as Latin America, Southeast Asia, and Central Africa. This research gap may lead to knowledge bias and missed opportunities to discover novel pediatric therapeutics.

Furthermore, the preclinical antimicrobial support of the majority of plants is consistent with traditional uses. However, toxicity makes translation difficult, especially for *Xylopia aethiopica*, which is widely used for malaria, and coughs has antioxidant, anticancer, and antimicrobial properties [37]. There is a delicate balance between preclinical red flags that have not been tested in humans and a strong ethnopharmacological justification for its sub-chronic rat toxicity at 1000 mg/kg (28 days), which indicates hepatic/renal risks. Similarly, *Anchomanes difformis*, used for respiratory purposes, exhibits acute mouse toxicity (400 mg/kg, 7 days). Overall, in vitro*/*in vivo efficacy is superior, but safety flaws (such as a lack of clinical PK/PD data or pediatric extrapolation limits) require that low-toxicity candidates like *Cymbopogon citratus* be given priority.

The justification for highlighting these toxicities lies in the unique vulnerability of paediatric populations. Children have immature detoxification systems and lower body weight, making them more susceptible to toxic substances [71]. This underscores the importance of conducting dose–response studies, long-term toxicity evaluations, and age-specific safety assessments, none of which are adequately addressed in the current literature. All findings in Table 5 derive from preclinical models: predominantly in vivo rodent acute/subchronic assays (mice/rats, n = 16 entries) measuring clinical signs/mortality, with two in vitro brine shrimp lethality tests (entries 7,13) as preliminary screens. No in vitro antimicrobial assays (e.g., MIC on respiratory pathogens) or clinical data are detailed, limiting the strength to Levels 3–4 on standard evidence pyramids.​Additionally, most of the reviewed toxicity studies were preclinical (animal models or in vitro tests), which limits direct extrapolation to humans, especially infant populations. As a result, while ethnomedicinal use may appear safe through long-standing tradition, scientific validation must still be pursued to mitigate risk, especially with polyherbal preparations or chronic use.

**Table 5:** Pharmacological activities and toxicological evaluation of traditional medicinal plants (TMPs) landscape

No	References	Country	Scientific name	Common name	Plant part	Compounds	Pharmacological activities	Toxicological evaluation
1.	Rastogi et al. [32], Bala et al. [33]	India	*Aloe vera* (L.) Burm.f	Cape aloe	-	-	Anti-inflammatory	Test: In vivo (mice) Toxicity: Absent Extract: *A. vera* gel Dose (toxicity): 100 and 250 mg/Kg body weight of extract in mice after 30 days
2.	Singh et al. [34], Abdillah et al. [35]	India	*Momordica charantia* L	Bitter gourd	L	Luteolin-7-glucoside, rutin	Antimicrobial, anticancer	Test: In vivo (mice) Toxicity: Absent Extract: *M. charantia* fruit Dose (subshronic toxicity): 40, 80 and 320 mg/Kg bodyweight of the extract in mice for 28 days
3.	Mohammedi et al. [36]	Algeria	*Ammodaucus leucotrichus* L	Wooly cumin	Fruits (Oil)	Perillaldehyde, limonene, α-pinene	Anti-inflammatory, antimicrobial	Test: In vivo (mice) Toxicity: Present Extract: *A. leucotrichus* oil Dose (acute toxicity): 520–570 mg/Kg of the extract in mice after 14 days
4.	Oguntimehin et al. [37], Olanlokun et al. [38]	Nigeria	*Anchomanes difformis* (Blume) Engl	-	L	Phenolic compounds	Antimicrobial	Test: In vivo (mice) Toxicity: Present Extract: *A. difformis* root Dose (toxicity): 400 mg/Kg weight of the extract in mice after 7 days
5.	Oguntimehin et al. [37], Otunola & Afolayan, [39]	Nigeria	*Capsicum frutescens* L	Chilli pepper	L	Phenolic compounds	Anticancer	Test: In vivo (diabetic mice) Toxicity: Present Extract: Polyherbal containing *C. frutescens* Dose (toxicity): 500 mg/Kg weight of the extract in diabetic mice after 7 days
6.	Oguntimehin et al. [37], Assih et al. [40]	Nigeria	*Xylopia aethiopica* (Dunal) A.Rich	Eru-alamo (Yoruba)	R	Phenolic compounds	Antimicrobial, anticancer, antioxidant	Test: In vivo (rats) Toxicity: Present Extract: *X. aethiopica* leaves Dose (sub-chronic toxicity): 1000 mg/Kg weight of the extract in rats after 28 days
7.	Erukainure et al. [12], Folami et al. [41]	Nigeria	*Vernonia amygdalina* Delile	Bitter leaf	L	Acidic lipid, mucilage, pectin, lipids, polyphenols, alkaloids	Antioxidant	Test: In vivo (rats) Toxicity: Absent Extract: *V. amygdalina* leaves Dose (toxicity): 150, 300, 600, and 1200 mg/Kg weight of the extract in rats after 28 days
8.	G'massampou et al. [42], Roland et al. [43]	Togo	*Annona senegalensis* Pers	African custard-apple	R	Alkaloids, tannins, flavonoids, carbohydrates, saponosides and phenols	Anti-inflammatory, antimicrobial	Test: In vivo (rats) Toxicity: Absent Extract: *A. senegalensis* leaves Dose (Acute toxicity): 5000 mg/Kg weight of the extract in rats after 14 days
9.	Yunitrianti & Noviani [44], Maygusten et al. [45]	Indonesia	*Ayapana triplinervis* (Vahl.)	Water hemp	L	Flavonoids, phenols, alkaloids, flavonoids, terpenoids, tannins, saponins	Antioxidant	Test: In vitro (brine shrimp lethality) Toxicity: Present Extract: *A. triplinervis* leaf Dose (Brine Shrimp Lethality Test): 18.86 ppm weight of the extract in shrimp larvae after 24 h
10.	Teffo et al. [46], Moroole et al. [47]	South Africa	*Bulbine frutescens* (L.) Willd	Stalked bulbine	L, R	Tannins, steroids, terpenoids, saponins, coumarins, glycosides phlobatannins	Antioxidant, antimicrobial	Test: In vivo (rats) Toxicity: Absent Extract: Polyherbal containing *B. frutescens* Dose (acute toxicity): 5, 50, 300 or 2000 mg/Kg weight of the extract in rats after 14 days
11.	Elansary et al. [48], Shaikh et al. [49]	Egypt	*Citrullus colocynthis* L	Bitter apple	Fr (coat)	Phenylalanine, Hydroxycaffeic acid 3,4-dihydroxyphenylacetic acid, 6-Hydroxy-4-methylcoumarin	Antioxidant, antimicrobial	Test: In vivo (rats) Toxicity: Present Extract: *C. colocynthis* fruit pulp Dose (acute toxicity): 1000 mg/Kg weight of the extract in rats after 4 days
12.	Sabir et al. [50], Murugan et al. [51]	Parkistan	*Curcuma longa* L	Turmeric	Rh	Curcumin, curcumin-O-glucuronide, Valoneic acid bilactone, Demethoxycurcumin, Isorhamnetin	Antioxidant	Test: In vivo (rats) Toxicity: Absent Extract: Standardized extract of *C. longa* Dose (sub-chronic toxicity): 250, 500, and 1000 mg/Kg weight of the extract in rats after 90 days
13.	Alzobaay & Kadhim [52], Xavier, et al. [53]	Iraq	*Cymbopogon citratus* (DC.) Stapf	Lemon grass	L	Alkaloids, glucosides, phenols, saponins, flavonoids, tannins, terpenoids, resins	Antibacterial	Test: In vivo (rats) Toxicity: Absent Extract: *C. citratus* oil Dose (acute toxicity): 2000 mg/Kg weight of the extract in rats after 14 days
14.	Edziri et al. [54], Yahiaoui et al. [55]	Tunisia	*Fumaria officinalis* L	Fumitory	Ap	Flavonoids, phenolic compounds	Antioxidant	Test: In vivo (mice) Toxicity: Present Extract: *F. officinalis* aerial parts (alkaloids) Dose (sub-acute toxicity): ≥ 500 mg/Kg weight of the extract in mice after 28 days
15.	Manandhar et al. [56], Tibuhwa, 2016 [57]	Nepal	*Oxalis corniculata* L	Sleeping beauty	L	-	Antimicrobial	Test: In vitro (brine shrimp) Toxicity: Present (mild toxicity) Extract: *Oxalis corniculata* whole plant Dose:156 µg/ml weight of the extract in shrimp larvae after 24 h
16.	Nounagnon et al. [13], Builders et al. [58]	Benin	*Parkia biglobosa* (Jacq) benth	African locust bean	L, B	Anthocyanins,flavonoids, glucosides, saponins, tannins,	Antioxidant, antimicrobial	Test: In vivo (rats) Toxicity: Present Extract: *P. biglobosa* stem bark Dose (toxicity): 5000 mg/Kg weight of the extract in rats after 14 days
17.	Rani & Giri [59], Mishra et al. [60], Mostafa et al. [61]; Jahromi et al. [62]	India Saudi Arabia	*Punica granatum* L	Pomegranate	P; L	Tannin, phlobatannis, anthroquinones, saponins, protein, glycosides, phlobatannins, alkaloids	Anticancer, antibacterial	Test: In vivo (mice) Toxicity: Absent Extract: *P. granatum* peel Dose (toxicity): 0.5, 1.9 and 7.5 mg/Kg weight of the extract in mice after 22 days
18.	Silva et al. [63], Al-azem et al. [64]	Brazil	*Syzygium aromaticum* (L.) Merr. & L.M.Perry	Clove	F	-	Antifungal	Test: In vivo (rats) Toxicity: Present Extract: *S. aromaticum* oil Dose (toxicity): 0.125, 500 mg/Kg weight of the extract in rats after 7 days ()
19.	Bonsou et al. [65]	Cameroon	*Tetrapleura tetraptera* (Schumm. & Thonn.) Taub	Aidan (Yoruba)	Fr	-	Anticancer	Test: In vivo (rats) Toxicity: Present Extract: *T. tetrapleura* Dose (sub-chronic toxicity): 500, 1000 mg/Kg weight of the extract in rats after 28 days
20.	Osman et al. [66], Mostafa et al. [39, 61]	Sudan Saudi Arabia	*Zingiber officinales* Roscoe	Ginger	Rh	-	Anticancer [66],antibacterial	Test: In vivo (diabetic rats) Toxicity: Absent Extract: Polyherbal containing *Z. officinales* Dose (toxicity): at 500 mg/Kg weight of the extract in diabetic rats after 7 days

^*^(-) not specified, *Ap* Aerial parts, *B* Bark, *Bu* Bulb, *L* Leaf, *P* Peel, *Rh* Rhizome, *R* Root, *F* Flower, *Fr* Fruit

## Conclusion and recommendation

This literature review found that medicinal plants are beneficial for a child’s well-being. Fifty-four (54) medicinal plants were reported to address respiratory and gastrointestinal issues, bulging fontanelles, and skin problems, among others. The plant part commonly used was the leaves and prepared as a decoction. The plants exhibited antimicrobial, antifungal, anti-inflammatory, and anticancer activities, which justify their uses in traditional medicine. However, case reports and toxicological studies indicate the potential threat they pose to children due to their toxicity. This may be due to a lack of proper training from government institutions for Traditional health practitioners, which compels them to operate in silos. Furthermore, ethnobotanical research in many regions is recommended to create a comprehensive database of the medicinal plants utilized in traditional medical practices. Further, safety evaluations are needed to support their use in children. In future research, plant extracts should be standardized and dosages that are safe and suitable for children should be established. To understand how these plants produce their antimicrobial, anti-inflammatory, and antioxidant effects, mechanistic research is required. Due to rats' faster metabolism, in vivo murine data overestimate safety margins. Additionally, pediatric factors such as immature Phase I/II enzymes, reduced glomerular filtration (50–70% adult levels at birth), and higher per-kg dosing norms are ignored. Moreover, brine shrimp assays produce false-positive results for cytotoxins unrelated to human systemic toxicity, and they show poor correlation (r < 0.6) with mammalian LD50 values. These cannot accurately forecast pediatric risks from traditional medicinal plants, where child dosing is common but non-standardized, without bridging studies like juvenile animal models. Human-relevant in vitro and microdosing trials ought to be the focus of future research.

## Data Availability

The article included the original contributions presented in the study. Further inquiries can be directed to the corresponding author.
